# Body Composition, Serum Concentrations of Androgens and Insulin Resistance in Different Polycystic Ovary Syndrome Phenotypes

**DOI:** 10.3390/jcm9030732

**Published:** 2020-03-09

**Authors:** Aleksandra Maria Polak, Agnieszka Adamska, Anna Krentowska, Agnieszka Łebkowska, Justyna Hryniewicka, Marcin Adamski, Irina Kowalska

**Affiliations:** 1 Department of Internal Medicine and Metabolic Diseases, Medical University of Białystok, 15-276 Białystok, Poland; alexandra_1991@op.pl (A.M.P.); a.krentowska@gmail.com (A.K.); a.lebkowska@wp.pl (A.Ł.); irinak@poczta.onet.pl (I.K.); 2 Department of Endocrinology, Diabetology and Internal Medicine, Medical University of Białystok, 15-276 Białystok, Poland; justynapliszka@gmail.com; 3 Faculty of Computer Science, Bialystok University of Technology, 15-351 Białystok, Poland; m.adamski@pb.edu.pl

**Keywords:** body composition, insulin resistance, androgens, PCOS phenotypes

## Abstract

Insulin resistance and hyperandrogenemia observed in polycystic ovary syndrome (PCOS) are associated with metabolic disturbances and could be connected with body composition pattern. To date, several studies defining the parameters of body composition using dual energy X-ray absorptiometry (DXA) method in the group of PCOS patients have been published, however, without the analysis in different phenotypes. The aim of the present study was to investigate the relationships between serum androgens concentration, insulin resistance and distribution of fat mass using DXA method in various PCOS phenotypes according to the Rotterdam criteria. We examined 146 women: 34 (38%) had PCOS phenotype A, 20 (23%) phenotype B, 20 (23%) phenotype C and 15 (16%) phenotype D (with mean age of each phenotype 25 years), and 57 control subjects (mean age of 25.5 years). Homeostasis model assessment of insulin resistance (HOMA-IR) was calculated. Serum concentrations of testosterone, androstenedione and dehydroepiandrosterone sulfate (DHEA-S) were assessed and free androgen index (FAI) was calculated. In phenotypes A, B and C, we observed higher FAI in comparison to the control group (all *p* < 0.01). Serum concentrations of androstenedione and DHEA-S were higher in phenotypes A and C in comparison to the control group (all *p* < 0.01). However, only in phenotype A we found higher visceral adipose tissue (VAT) mass and android/gynoid ratio (A/G ratio) in comparison to the control group (all *p* < 0.01). In phenotype A, we observed connection of VAT with FAI (*r* = 0.58, *p* < 0.01). Accordingly, A/G ratio was related with FAI in all phenotypes (all *p* < 0.05). Additionally, in phenotype C, A/G ratio was related to serum concentrations of DHEA-S and androstenedione (*r* = 0.46, *p* = 0.03; *r* = 0.53, *p* = 0.01, respectively). We also found connections of HOMA-IR with VAT and A/G ratio in all phenotypes (all *p* < 0.05). Women with phenotype A had higher amount of VAT and A/G ratio in comparison to the control group. Serum concentration of androgens and insulin resistance are connected with VAT and A/G ratio in normoandrogenic and hyperandrogenic PCOS phenotypes.

## 1. Introduction

Polycystic ovary syndrome (PCOS) is a common endocrinopathy in women of reproductive age, with a prevalence of 6–20% according to the criteria used [1]. Most women with PCOS are also characterized by metabolic abnormalities like abdominal obesity or insulin resistance, which form the risk factors for metabolic syndrome [2]. A number of studies have indicated that insulin resistance plays a crucial role in the pathogenesis of polycystic ovary syndrome [3]. Insulin acts synergistically with luteinizing hormone (LH), leading to increased production of androgens in the ovarian theca cells [4]. Hyperandrogenemia includes elevated serum concentrations of total and free testosterone, androstenedione and dehydroepiandrosterone sulfate (DHEA-S). Previous data have shown that hyperandrogenemia may affect the distribution of adipose tissue in PCOS patients [5]. Additionally, it has been reported that increased serum testosterone levels in PCOS women are associated with excess of visceral fat amount [5], as well as with insulin resistance and more frequent occurrence of impaired glucose tolerance [3].

In the Rotterdam Consensus, it was defined that in order to diagnose PCOS, at least two of the following criteria have to be fulfilled: oligoovulation and/or anovulation, clinical and/or biochemical hyperandrogenism, and polycystic ovarian morphology in transvaginal ultrasound [6]. The Rotterdam criteria for PCOS recognize four clinical phenotypes of the syndrome. The most prevalent phenotype is the classic form [7], which meets all three current criteria for PCOS: clinical and/or biochemical hyperandrogenism (HA), menstrual dysfunction (oligo/amenorrhea) (Oligo) and polycystic ovarian morphology (PCOM)-phenotype A (Oligo + HA + PCOM). Phenotype B (HA + Oligo) and phenotype C (HA + PCOM) are less frequent. The Rotterdam criteria also recognize a fourth phenotype, D, which is defined by oligomenorrhea, polycystic ovarian morphology in ultrasound and normal androgen levels (Oligo + PCOM) [8]. An increased incidence of metabolic disorders is observed among women with phenotypes A, B and C [9], whereas phenotype D is probably characterized by fewer metabolic abnormalities [1]. However, not all published data confirm this hypothesis [1].

To date, several studies defining the parameters of body composition using dual energy X-ray absorptiometry (DXA) method in the group of PCOS patients have been published [10,11,12], however, without the division into phenotypes. Magnetic resonance imaging is considered the gold standard in the assessment of fat distribution (visceral and subcutaneous adipose tissue). However, this technique requires advanced equipment and highly qualified staff. It has been shown that visceral obesity might be detected at an early stage by DXA. Moreover, due to high reproducibility of this method, repeated measurements might be performed in the same patient to monitor changes in body composition over time [13].

As it was mentioned previously, hyperandrogenemia is connected with adverse metabolic parameters, therefore, we hypothesized that women with phenotypes characterized by elevated serum concentration of androgens (phenotypes A, B and C) presented insulin resistance and adverse fat distribution compared with those with normal serum level of androgens (phenotype D). Therefore, the aim of the present study was to investigate the relationships between serum androgen concentrations, insulin resistance and distribution of fat mass using the DXA method in various PCOS phenotypes.

## 2. Materials and Methods

### 2.1. Subjects

A prospective, cross-sectional study was conducted between March 2018 and June 2019. The study group consisted of 146 women: 89 patients with PCOS divided into four phenotypes (phenotype A, B, C, D with mean age of 25 years), and 57 control women (mean age of 25.5 years). PCOS women were patients treated in the Department of Endocrinology, Diabetology and Internal Medicine and the Department of Internal Medicine and Metabolic Diseases, Medical University of Białystok. The control group was recruited from students who met exclusion criteria and met the following criteria: they were normoandrogenic, without hirsutism, had a history of regular, ovulatory menstrual cycles and morphologically normal ovaries on ultrasound. The diagnosis of PCOS was made according to the 2003 Rotterdam ESHRE/ASRM PCOS Consensus Workshop Group diagnostic criteria. We defined PCOS by the presence of at least two out of three criteria: clinical and/or biochemical hyperandrogenism, oligo/anovulation, and polycystic ovaries in ultrasound (>12 follicles measuring 2–9 mm in diameter or ovarian volume >10 mL in at least one ovary) [6]. The phenotypes of PCOS (A, B, C, D) were classified according to Rotterdam criteria described in the Introduction section [6]. Exclusion criteria included: other conditions causing menstrual irregularity and/or hyperandrogenism (i.e., hyperprolactinemia, Cushing’s syndrome (based on history taking and physical examination), late-onset congenital adrenal hyperplasia (for this purpose, we determined the serum levels of 17-hydroxyprogesterone), hypothyroidism and hyperthyroidism, pregnancy (appropriate test was performed) and breastfeeding, type 1 or type 2 diabetes, chronic or acute infection (within the previous 30 days), any other serious medical problem, hormonal contraception and/or anti-androgen therapy (within the previous 6 months), and the use of medications for obesity, hyperglycemia, dyslipidemia or hypertension. All the patients participating in the study were Caucasians. The study was approved by the Institutional Review Board (Ethics Committee of the Medical University of Białystok, Białystok, Poland; approval no. R-I-002/127/2018) and was concordant with the Declaration of Helsinki. All the procedures were performed in accordance with the relevant guidelines and regulations. All women participated in the study voluntarily and gave their written informed consent for inclusion. All the procedures were explained to the participants in detail before the beginning of the study.

### 2.2. Study Protocol

All women underwent physical examination. Clinical hyperandrogenism-hirsutism (defined as more than eight points in the modified Ferriman-Gallwey score) [14] and presence of acne were evaluated. Oligo/amenorrhea and anovulation were defined as fewer than six menses during the previous year.

BMI was calculated as body weight in kilograms divided by height in meters squared (kg/m^2^). Waist circumference was measured in the standing position, at the smallest circumference between the rib cage and the iliac crest. Systolic and diastolic blood pressure was recorded. Transvaginal ultrasound was performed in all women by the same gynecologist with a 5–9 MHz transvaginal transducer (Voluson 730 Expert GE Healthcare) in the early follicular phase. Ovarian volume was calculated using the simplified formula for a prolate ellipsoid [15].

In the morning, blood samples were obtained between the 3rd and 6th day of the cycle or independently of cycle phase in the presence of amenorrhea, at least 3 months from the last menses. Oral glucose tolerance test with 75 g of glucose was performed in all subjects to exclude diabetes.

### 2.3. Biochemical Analyses

Fasting plasma glucose and serum insulin concentrations, as well as plasma concentrations of glucose and serum levels of insulin two hours after the ingestion of 75 g of glucose were determined. Plasma glucose concentrations were assessed by the hexokinase method, and plasma lipid concentrations (total cholesterol (TC), high-density lipoprotein cholesterol (HDL-C), triglycerides (TG)) were measured by enzymatic colorimetric method (Cobas c111, Roche Diagnostic Ltd., Switzerland). Plasma low-density lipoprotein cholesterol (LDL-C) was calculated with the Friedewald’s formula. Serum insulin concentrations were assessed with the immunoradiometric method (DIAsource ImmunoAssays S.A., Belgium) (minimum detectable concentration (MDC)—1 µIU/mL; intra-assay coefficient of variation (CV)—below 2.2%, inter-assay CV—below 6.5%,). There is no cross-reaction between human and animal proinsulins in this method.

Serum follicle-stimulating hormone (FSH) and luteinizing hormone (LH) levels were determined with immunoradiometric method (DIAsource ImmunoAssays S.A., Belgium) (LH: intra-assay CV—below 3.9%, inter-assay CV—below 8%; FSH: intra-assay CV—below 2%, inter-assay CV—below 4.4%). Concentrations of total testosterone were measured by radioimmunoassay (DIAsource ImmunoAssays S.A., Belgium) (MDC—0.05 ng/mL, intra-assay CV—3.3%, inter-assay CV—4.8%). Serum sex hormone–binding globulin (SHBG) concentrations were assessed with immunoradiometric method (ZenTech, Angleur, Belgium) (intra-assay CV—below 5.2%, inter-assay CV—below 5.8%). Serum concentrations of DHEA-S and androstenedione were measured with radioimmunoassay (DIAsourceImmunoAssays S.A., Belgium) (MDC for DHEA-S—1.23 µg/dL, for androstenedione—0.03 ng/mL; intra-assay and inter-assay CV for DHEA-S—3.6% and 6.5%, for androstenedione—3.2% and 5.9%).

### 2.4. Calculations

Homeostasis model assessment of insulin resistance (HOMA-IR) was calculated as fasting insulin (µIU/mL) x fasting plasma glucose (mmol/L)/22.5 [16]. Free androgen index (FAI) was calculated according to the formula: serum total testosterone (nmol/L) × 100/SHBG (nmol/L) [14].

### 2.5. Body Composition Analysis

Body composition analysis was conducted using DXA (GE Healthcare, Chicago, IL, USA, Lunar iDXA) by qualified physicians at the Clinical Research Centre, Medical University of Białystok. The equipment was calibrated before every examination. The patients were positioned on the examination table in a supine position, with their feet secured together with an adjustable strap and hands lying flat adjacent to the sides of the body. Each examination took approximately 8 min. On the basis of the scans, CoreScan software estimated mass of visceral adipose tissue (VAT) within the android region. Additionally, android/gynoid ratio (A/G ratio) was calculated. DXA assessed fat mass with the precision (coefficient of variation) of 2.0% and 8.0%, respectively.

### 2.6. Statistical Analysis

The statistical analysis for the present study was performed with the Statistica package (Statistica 13.3, Statsoft, Cracow, Poland). All analyzed variables were tested for normality of distribution with the Shapiro–Wilk test. Due to non-normal distribution, all values were expressed as median (interquartile range). Differences between the studied groups were assessed with non-parametric Kruskal–Wallis test with post-hoc multiple comparisons of mean ranks of all pairs of groups. Correlation analysis was performed using the Spearman test. Afterwards, multivariate regression analysis was performed to investigate independent relationships. A *p*-value < 0.05 was considered statistically significant.

## 3. Results

The clinical characteristics of the studied groups are presented in Table 1. In PCOS group, 34 (38%) women had phenotype A, 20 (23%) women had phenotype B, 20 (23%) women presented phenotype C and 15 (16%) were diagnosed with phenotype D. The groups were similar in terms of age and BMI (all *p* > 0.05) (Table 1).

Serum concentrations of total testosterone were significantly higher in phenotype A (*p* < 0.01 in post-hoc analysis) and C (*p* < 0.01 in post-hoc analysis) in comparison to the control group. Similarly, a higher level of total testosterone was observed in phenotypes A, B and C in comparison to phenotype D (in post-hoc analysis *p* < 0.01; *p* = 0.01; *p* < 0.01; respectively). We noticed lower serum concentration of SHBG in phenotype A and B in comparison to the controls (in post-hoc analysis both *p* < 0.01). In phenotypes A, B and C, FAI was higher in comparison to the controls (in post-hoc analysis all *p* < 0.01). Accordingly, we observed higher FAI in phenotype A in comparison to phenotype D (*p* < 0.01). Serum concentrations of DHEA-S were higher in phenotype A and C in comparison to the healthy women (in post-hoc analysis both *p* < 0.01). Similarly, we noticed higher serum concentrations of androstenedione in phenotype A and C in comparison to the control group (in post-hoc analysis both *p* < 0.01). We did not observe differences in HOMA-IR between the studied groups (*p* = 0.25), however, fasting glucose was higher in phenotype A vs. C (in post-hoc analysis *p* = 0.04) (Table 1).

**Figure 1:**
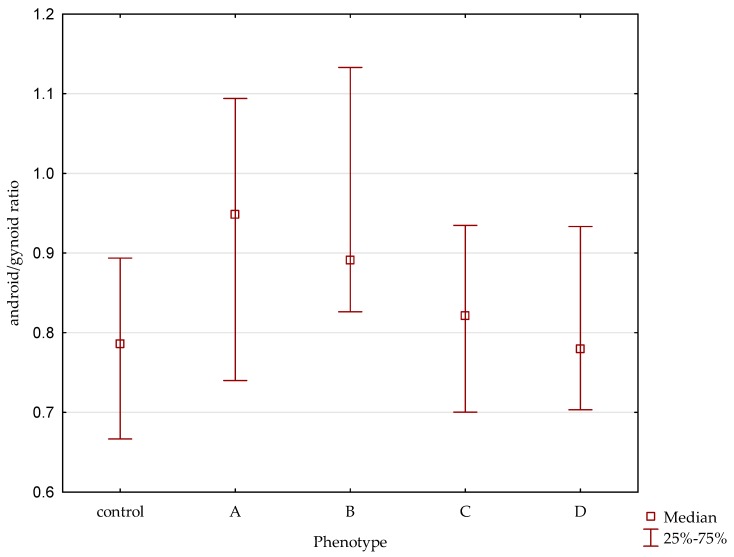
Android/gynoid ratio in different PCOS phenotypes and the control group.

DXA analysis revealed higher VAT mass (in post-hoc analysis *p* = 0.04) and A/G ratio (in post- hoc analysis *p* = 0.01) in phenotype A than in the control group (Figure 1).

In phenotype A, we observed relationships between FAI and VAT (*r* = 0.58, *p* < 0.01). We also found connections of HOMA-IR with VAT in phenotypes A, B, C and D (all *p* < 0.05). We did not find significant relationships of serum concentrations of DHEA-S and androstenedione with VAT estimated with DXA in phenotypes A, B, C and D (all *p* > 0.05) (Table 2).

In phenotypes A, B, C and D, we observed relationships between FAI and A/G ratio (all *p* < 0.01). We also found connections of HOMA-IR with A/G ratio in phenotypes A, B, C and D (all *p* < 0.05). However, only in phenotype C, serum concentrations of DHEA-S and androstenedione were connected with A/G ratio (*r* = 0.46, *p* = 0.03; *r* = 0.53, *p* = 0.01, respectively). We found no correlation of serum concentration of androstenedione and DHEA-S with A/G ratio estimated with DXA in phenotype A, B and D (all *p* > 0.05) (Table 3).

We found relationships between FAI and HOMA-IR in phenotype A (*r* = 0.40, *p* = 0.01), phenotype B (*r* = 0.47, *p* = 0.03) and phenotype C (*r* = 0.66, *p* = 0.001), but not in phenotype D (*r* = 0.36, *p* = 0.18).

In the entire group, multiple regression analysis showed that FAI (β = 0.33, *p* < 0.01) and HOMA-IR (β = 0.36, *p* < 0.01) were significantly associated with A/G ratio and there was no significant interaction with phenotypes. Additionally, in the entire group, multiple regression analysis showed that FAI (β = 0.37, *p* < 0.01) and HOMA-IR (β = 0.52, *p* < 0.01) were significantly associated with VAT mass and there was no significant interaction with phenotypes.

In the control group, we found no correlation between HOMA-IR, serum concentrations of androstenedione, DHEA-S and VAT estimated with DXA (all *p* > 0.05) (Table 2). We observed relationships between A/G ratio and FAI (*r* = 0.26, *p* = 0.04) in the control group (Table 3).

## 4. Discussion

In our study, we demonstrated the relationships of serum concentrations of different androgens and HOMA-IR with body composition estimated with DXA in different phenotypes of PCOS. In phenotype A, we observed higher VAT amount, as well as A/G ratio and FAI in comparison to the control group, and a connection between FAI and VAT and A/G ratio in this phenotype. Previous studies have shown contrasting results of fat content in PCOS women [12,17,18]. In some studies, increased abdominal fat was observed in overweight and lean PCOS women in comparison to controls [18], whereas in others, fat mass in trunk and arms were significantly higher in patients with PCOS vs. control [12]. In the cited study, FAI positively correlated only with fat mass in arms in women with PCOS [12]. However, they did not examine various PCOS phenotypes, as we did. In one study, there were no differences in fat distribution in DXA method between phenotypes. However, the authors found that A/G ratio was connected positively with HOMA-IR and negatively with insulin sensitivity index [19]. Previous studies have shown that VAT is metabolically more active than subcutaneous adipose tissue, and that the increased amount of VAT is associated with higher risk of metabolic disturbances, e.g., hypertension, dyslipidemia, insulin resistance and type 2 diabetes [20]. It has also been observed that fat distribution is altered in PCOS patients and that this group presents greater tendency to increased VAT accumulation in comparison to the general population [17]. Moreover, it has been shown that VAT is associated with insulin resistance and increased metabolic risk in PCOS women [21], and that increased concentrations of androgens are connected with abdominal fat deposition [22]. Therefore, our results confirmed that phenotype A could be considered a phenotype with increased risk of obesity, type 2 diabetes, coronary heart disease and other metabolic disorders [23], and it may be related to significantly higher FAI in this group of patients. However, prospective studies are needed to confirm this hypothesis.

It is unclear whether hyperandrogenic PCOS phenotypes are at an increased cardiovascular risk in comparison to normoandrogenic phenotype. In the present study, relationships between serum concentration of androgens and body distribution in various PCOS phenotypes were observed. We revealed relationships between FAI and A/G ratio in phenotypes with hyperandrogenism and normoandrogenic phenotype. We also observed that serum concentrations of DHEA-S and androstenedione were connected with A/G ratio in phenotype C. Furthermore, we demonstrated the association of HOMA-IR and FAI with A/G ratio in all phenotypes. Interestingly, in phenotypes C and D, the relationship between FAI and VAT almost reached statistical significance. Therefore, we could not exclude connections between fat distribution and FAI in those phenotypes. Therefore, it seems that women without elevated serum androgens concentration are not protected from metabolic disturbances. On the contrary, Carmina et al. [18] did not find any correlation between fat parameters and serum testosterone levels in PCOS patients. However, they did not study various PCOS phenotypes. Our observation can be supported by the fact that insulin resistance in PCOS women is related to excessive serine phosphorylation of the insulin receptor 1 (IRS-1) [24], and serine phosphorylation modulates the activity of the key regulatory enzyme of androgen biosynthesis, P450c17 [25].

In our study, we reported a significant difference among the four studied phenotypes in terms of total testosterone levels. However, we found that in phenotype D, serum concentration of total testosterone was similar to control group and lower in comparison to other phenotypes. Our findings are in accordance with Jamil et al. [26], who also reported higher total testosterone levels in phenotype A, B and C than in phenotype D. Additionally, Yilmaz et al. reported that phenotype D was more similar to the control group than the other PCOS phenotypes [27]. Those results suggest that PCOS patients are not a homogenous group in relation to androgen excess. Previous studies [2,26,28] confirmed that metabolic abnormalities are less severe in normoandrogenic women with PCOS in comparison to phenotypes with hyperandrogenism. However, based on our data, we could not confirm that phenotype D is characterized by milder endocrine and metabolic abnormalities than other PCOS phenotypes.

The limitation of the present study is a relatively small number of participants representing different PCOS phenotypes, however, they are very well characterized. Another limitation is the use of HOMA-IR to estimate insulin resistance. The gold standard in the assessment of whole-body insulin sensitivity is hyperinsulinemic euglycemic clamp, however, it is time-consuming and difficult to perform [29]. Other methods of insulin sensitivity assessment could be minimal model S_1_ and indirect indices calculated from OGTT [30]. HOMA-IR takes into account fasting glucose and insulin levels and only reflects hepatic insulin sensitivity [31]. Therefore, the correlation between HOMA-IR and M index derived from the clamp is only moderate [29]. It should also be emphasized that concentrations of total testosterone were measured by RIA. The currently recommended method, considered a gold standard in the assessment of testosterone concentrations, is liquid chromatography-tandem mass spectrometry (LC-MS), however, its use is limited by high costs of the technique.

## 5. Conclusions

In conclusion, women with phenotype A have a higher amount of VAT and A/G ratio in comparison to the control group, therefore, metabolic disturbances could be more pronounced in this phenotype. Serum concentration of androgens and insulin resistance are connected with VAT and A/G ratio in normoandrogenic and hyperandrogenic PCOS phenotypes.

## Figures and Tables

**Table 1:** Clinical and biochemical characteristics of the studied groups.

	Control Group (*n* = 57)	Phenotype A (*n* = 34)	Phenotype B (*n* = 20)	Phenotype C (*n* = 20)	Phenotype D (*n* = 15)	*p* Value
Age (years)	25 (23–28)	24 (22–27)	24 (23–27.5)	24 (21.5–27.5)	26 (22–28)	0.60
BMI (kg/m^2^)	22.4 (21.7–24.3)	23.7 (21.1–29.4)	24.9 (22.2–29.5)	23.1 (21.6–25.2)	23.4 (20.5–27.1)	0.60
WC (cm)	80 (74–84)	80 (73–96)	81.5 (77.5–97)	78.5 (73.5–89)	82 (69–91)	0.72
Ferriman-Gallwey score	3 (2–5)	9 (4–12) ^1,2^	11 (9–15) ^3,6^	9 (3–11) ^4,5^	1 (1–5) ^2,3,5^	<0.01
FSH (IU/L)	5.40 (4.4–6.3)	5.61 (3.62–6.39)	4.76 (3.91–6.02)	5.67 (5.14–6.57)	5.19 (4.34–6.6)	0.41
LH (IU/L)	3.70 (2.7–4.7)	4.66 (3.1–7.2)	3.66 (2.57–4.56)	3.71 (2.95–4.91)	4.42 (3.58–5.95)	0.07
TT (ng/mL)	0.56 (0.42–0.69)	0.72 (0.63–0.94) ^1,2^	0.78 (0.59–0.88) ^3^	0.80 (0.65–0.89) ^4,5^	0.51 (0.41–0.59)	<0.01
SHBG (nmol/L)	66.9 (54.6–92.5)	43.2 (27.2–51.8) ^1^	34.7 (25.8–86.6) ^6^	56.3 (36.7–73.4)	57.5 (51.5–79.7)	<0.01
FAI	2.70 (1.7–3.8)	6.18 (4.34–9.73) ^1,2^	5.38 (2.69–8.94) ^4^	4.65 (3.19–6.59) ^6^	2.58 (2.04–3.5)	<0.01
Androstenedione (ng/mL)	3.10 (2.49–4.04)	4.60 (3.3–5.1)^1^	3.73 (3.2–4.62)	4.68 (3.26–5.85) ^4^	3.43 (2.9–4.95)	<0.01
DHEA-S (ug/dL)	230.1 (185.6–338)	300.3 (256.9–368.6) ^1^	287.1 (222.8–400.6)	358.6 (241.9–441.6) ^4^	238 (201.5–301.4)	<0.01
Glucose 0′ OGTT (mg/dL)	92 (88–97)	95 (90–100) ^7^	96.5 (91–100)	90 (84–92)	90 (87–94)	0.04
Glucose 120′ OGTT (mg/dL)	91 (75–101)	98 (86–121)	96.5 (85.5–111)	85 (78–98)	83 (77–95)	0.03
Insulin 0′ OGTT (uIU/mL)	8.80 (7.2–11.6)	10.60 (7.5–14.8)	9.81 (6.6–14.2)	8.36 (7.1–10.0)	8.20 (6.8–13.5)	0.38
Insulin 120′ OGTT (uIU/mL)	27.1 (18.7–38)	41 (25.5–67.9)	29.6 (25.8–46.6)	23.8 (17.2–40.3)	29.2 (19.2–57.1)	0.04
HOMA-IR	2.06 (1.64–2.9)	2.60 (1.91–3.49)	2.35 (1.45–3.61)	1.85 (1.46–2.3)	1.92 (1.46–2.73)	0.25
Total cholesterol (mg/dL)	171 (149–195)	172 (157–199)	169.5 (160–182)	168 (157–179.5)	174 (140–193)	0.80
HDL-cholesterol (mg/dL)	63 (57–75)	67 (49–75)	60.5 (50.5–69.5)	69.5 (59–77.5)	69 (51–79)	0.38
LDL-cholesterol (mg/dL)	90 (76–106)	96.6 (81.2–110.6)	91.3 (83.6–103)	86.6 (68.8–91.6)	90.8 (72–104)	0.28
TG (mg/dL)	59 (42–81)	67 (49–92)	68 (51.5–102)	60 (47.5–83.5)	60 (50–76)	0.30
VAT mass (g)	168 (68–336)	242 (125–897) ^1^	220 (88–667)	157 (57–356)	219 (118–420)	0.01
A/G ratio	0.79 (0.67–0.89)	0.94 (0.74–1.09) ^1^	0.88 (0.78–1.13)	0.82 (0.70–0.93)	0.76 (0.69–0.93)	0.005

Values are expressed as median (interquartile range): ^1^ *p* < 0.05 phenotype A vs. control; ^2^ *p* < 0.05 phenotype A vs. phenotype D; ^3^ *p* < 0.05 phenotype B vs. phenotype D; ^4^ *p* < 0.05 phenotype C vs. control; ^5^ *p* < 0.05 phenotype C vs. phenotype D; ^6^ *p* < 0.05 phenotype B vs. control; ^7^ *p* < 0.05 phenotype A vs. phenotype C. BMI: body mass index; WC: waist circumference; TT: total testosterone; DHEA-S: dehydroepiandrosterone sulfate; TG: triglycerides; OGTT: oral glucose tolerance test; FSH: follicle-stimulating hormone; LH: luteinizing hormone; FAI: free androgen index; SHBG: sex hormone binding globulin; HOMA-IR: homeostasis model assessment of insulin resistance; TSH: thyroid-stimulating hormone; VAT: visceral adipose tissue; A/G ratio: android/gynoid ratio.

**Table 2:** Relationship of HOMA-IR and serum concentration of androgens with VAT estimated with DXA method in the studied groups.

	Control Group (*n* = 57)	Phenotype A (*n* = 34)	Phenotype B (*n* = 20)	Phenotype C (*n* = 20)	Phenotype D (*n* = 15)
HOMA-IR	*r* = 0.12, *p* = 0.37	*r* = 0.61, *p* < 0.01 *	*r* = 0.70, *p* < 0.01 *	*r* = 0.51, *p* = 0.02 *	*r* = 0.57, *p* = 0.03 *
TT (ng/mL)	*r* = 0.14, *p* = 0.27	*r* = 0.20, *p* = 0.86	*r* = 0.01, *p* = 0.94	*r* = 0.13, *p* = 0.56	*r* = 0.05, *p* = 0.85
FAI	*r* = 0.22, *p* = 0.08	*r* = 0.58, *p* < 0.01 *	*r* = 0.38, *p* = 0.10	*r* = 0.44, *p* = 0.05	*r* = 0.50, *p* = 0.06
Androstenedione (ng/mL)	*r* = 0.18, *p* = 0.18	*r* = 0.09, *p* = 0.58	*r* = 0.11, *p* = 0.63	*r* = 0.22, *p* = 0.43	*r* = 0.11, *p* = 0.63
DHEA-S (ug/dl)	*r* = 0.09, *p* = 0.50	*r* = 0.12, *p* = 0.48	*r* = 0.05, *p* = 0.84	*r* = 0.21, *p* = 0.35	*r* = 0.27, *p* = 0.34

Data are derived from Spearman correlation coefficient. The level of significance was accepted at * *p* < 0.05. VAT: visceral adipose tissue; DXA: dual energy X-ray absorptiometry; HOMA-IR: homeostasis model assessment of insulin resistance; TT: total testosterone; FAI: free androgen index; DHEA-S: dehydroepiandrosterone sulfate.

**Table 3:** Relationship of HOMA-IR and serum concentration of androgens with A/G ratio estimated with DXA in the studied groups.

	Control Group (*n* = 57)	Phenotype A (*n* = 34)	Phenotype B (*n* = 20)	Phenotype C (*n* = 20)	Phenotype D (*n* = 15)
HOMA-IR	*r* = 0.12, *p* = 0.36	*r* = 0.53, *p* < 0.01 *	*r* = 0.53, *p* = 0.01 *	*r* = 0.50, *p* = 0.02 *	*r* = 0.58, *p* = 0.02 *
TT (ng/mL)	*r* = 0.05, *p* = 0.67	*r* = 0.16, *p* = 0.33	*r* = 0.08, *p* = 0.74	*r* = 0.51, *p* = 0.01	*r* = 0.06, *p* = 0.82
FAI	*r* = 0.26, *p* = 0.04 *	*r* = 0.53, *p* < 0.01 *	*r* = 0.50, *p* = 0.02 *	*r* = 0.61, *p* = 0.003 *	*r* = 0.52, *p* = 0.04 *
Androstendione (ng/mL)	*r* = 0.09, *p* = 0.48	*r* = 0.02, *p* = 0.89	*r* = -0.05, *p* = 0.83	*r* = 0.53, *p* = 0.01 *	*r* = -0.05, *p* = 0.83
DHEA-S (ug/dL)	*r* = 0.08, *p* = 0.55	*r* = 0.002, *p* = 0.48	*r* = 0.24, *p* = 0.30	*r* = 0.46, *p* = 0.03 *	*r* = 0.48, *p* = 0.06

Data are derived from Spearman correlation coefficient. The level of significance was accepted at * *p* < 0.05. A/G: android/gynoid ratio; HOMA-IR: homeostasis model assessment of insulin resistance; TT: total testosterone; FAI: free androgen index; DXA: dual energy x-ray absorptiometry; DHEA-S: dehydroepiandrosterone sulfate.
